# Supplementary material for: Risk factors for unfavorable outcome and impact of early post-transplant infection in solid organ recipients with COVID-19: A prospective multicenter cohort study
Source: PLoS One. 2021 Apr 29;16(4):e0250796. doi: 10.1371/journal.pone.0250796 (PMC8084252; doi:10.1371/journal.pone.0250796)
Supplement: S1 File — (DOCX) [file pone.0250796.s001.docx]

**S1 File. The COVIDSOT Working Team.**

*Lead author:* Elisa Cordero ([elisacorderom@gmail.com](mailto:elisacorderom@gmail.com))

*Virgen del Rocío University Hospital-IBiS, University of Seville, Seville, Spain*: Elisa Cordero, Jerónimo Pachón, Manuela Aguilar-Guisado, Judith Berastegui-Cabrera, Gabriel Bernal-Blanco, Pedro Camacho, Marta Carretero, José Miguel Cisneros, Juan Carlos Crespo, Miguel Angel Gómez-Bravo, Carmen Infante-Domínguez, Silvia Jiménez-Jorge, Laura Merino, Clara Rosso, Sonsoles Salto-Alejandre, Javier Sánchez-Céspedes, José Manuel Sobrino-Márquez. *Bellvitge University Hospital, Bellvitge Biomedical Research Institute-IDIBELL, University of Barcelona, Barcelona, Spain*: Jordi Carratalá, Nuria Sabé, Carme Baliellas, Oriol Bestard, Carles Diez, José Gonzàlez-Costello, Laura Lladó, Eduardo Melilli. *Puerta de Hierro University Hospital, Madrid, Spain*: Antonio Ramos-Martínez, Jorge Calderón-Parra, Ana Arias-Milla, Gustavo Centeno-Soto, Manuel Gómez-Bueno, Rosalía Laporta-Hernández, Alejandro Muñoz-Serrano, Beatriz Sánchez-Sobrino. *Clinic University Hospital-IDIBAPS, University of Barcelona, Barcelona, Spain*: Asunción Moreno, María Ángeles Marcos, Laura Linares, Marta Bodro, María Ángeles Castel, Frederic Cofán, Jordi Colmenero, Fritz Dieckmann, Javier Fernández, Dra. Marta Farrero, Miquel Navasa, Félix Pérez-Villa, Pedro Ventura. *Gregorio Marañón University Hospital, CIBERES, Madrid, Spain*: Maricela Valerio, Patricia Muñoz, Víctor Fernández-Alonso, Maria Olmedo-Samperio, Carlos Ortíz, Sara Rodríguez-Fernández, Maria Luisa Rodríguez-Ferrero, Magdalena Salcedo, Eduardo Zataraín. *Ramón y Cajal University Hospital, Madrid, Spain*: Pilar Martín-Dávila, Jesús Fortún-Abete, Juan Carlos Galán, Cristina Galeano-Álvarez, Francesca Gioia, Javier Graus, Sara Jiménez, Mario J. Rodríguez. *12 de Octubre University Hospital/i+12, CIBERCV, Madrid, Spain*: José María Aguado, Mario Fernández-Ruiz, Amado Andrés, Juan F. Delgado, Carmelo Loinaz, Francisco López-Medrano, Rafael San Juan. *Marqués de Valdecilla University Hospital-IDIVAL, University of Cantabria, Santander, Spain*: Carmen Fariñas, Francisco Arnaiz de las Revillas, Marta Fernández-Martínez, Ignacio Fortea-Ormaechea, Aritz Gil-Ongay, Mónica Gozalo-Marguello, Claudia González-Rico, Milagros Heras-Vicario, Víctor Mora-Cuesta. *La Fe University Hospital, Valencia, Spain*: Marino Blanes-Julia, Rosa Blanes-Hernández, Luis Almener-Bonet, María Isabel Beneyto-Castelló, Victoria Miguel Salavert-Lletí, Aguilera-Sancho-Tello, Amparo Solé-Jover. *Reina Sofía University Hospital-IMIBIC, Córdoba, Spain*: Elisa Vidal, Julián de la Torre-Cisneros, Rafael León, Álvaro Torres de Rueda, José Manuel Vaquero. *Virgen Macarena University Hospital-IBiS, Seville, Spain*: Zaira R. Palacios-Baena, Luis E. López-Cortés, David Gutiérrez-Campos, Marie-Alix Clement, Marta Fernández-Regaña, Inmaculada López-Hernández, Natalia Maldonado-Lizarazo, Ana Belén Martín-Gutiérrez, Rocío Valverde. *Badajoz University Hospital, Extremadura, Spain*: Román Hernández-Gallego, Elena García de Vinuesa-Calvo.
