# Supplementary material for: Risk factors for unfavorable outcome and impact of early post-transplant infection in solid organ recipients with COVID-19: A prospective multicenter cohort study
Source: PLoS One. 2021 Apr 29;16(4):e0250796. doi: 10.1371/journal.pone.0250796 (PMC8084252; doi:10.1371/journal.pone.0250796)
Supplement: S2 File — (DOCX) [file pone.0250796.s002.docx]

**S2 File. Institutional review board approval number of each participating center.**

Bellvitge University Hospital (C.I. PR116/20)

Puerta de Hierro University Hospital (C.I. 0683-N-20)

Clinic University Hospital (C.I. HCB/2020/0383)

Gregorio Marañón University Hospital (C.I. 142.20)

Ramón y Cajal University Hospital (C.I. 0683-N-20)

12 de Octubre University Hospital (C.I. 20/154)

Marqués de Valdecilla University Hospital (C.I. 2020.146)

La Fe University Hospital (C.I. 0683-N-20)

Reina Sofía University Hospital (C.I. 4638)

Badajoz University Hospital (C.I. 0683-N-20)
