# Supplementary material for: Risk factors for unfavorable outcome and impact of early post-transplant infection in solid organ recipients with COVID-19: A prospective multicenter cohort study
Source: PLoS One. 2021 Apr 29;16(4):e0250796. doi: 10.1371/journal.pone.0250796 (PMC8084252; doi:10.1371/journal.pone.0250796)
Supplement: S1 Table — (DOCX) [file pone.0250796.s003.docx]

**S1 Table. STROBE checklist.**

|  | **Item No** | **Recommendation** | **Manuscript location** |
| --- | --- | --- | --- |
| **Title and abstract** | 1 | (*a*) Indicate the study’s design with a commonly | Title and abstract |
|  |  | used term in the title or the abstract |  |
|  |  | (*b*) Provide in the abstract an informative and | Abstract |
|  |  | balanced summary of what was done and what |  |
|  |  | was found |  |
| **Introduction** |  |  |  |
| Background/rationale | 2 | Explain the scientific background and rationale | Introduction |
|  |  | for the investigation being reported |  |
| Objectives | 3 | State specific objectives, including any | Introduction, last |
|  |  | prespecified hypotheses | paragraph |
| **Methods** |  |  |  |
| Study design | 4 | Present key elements of study design early in the | Methods, first |
|  |  | paper | paragraph |
| Setting | 5 | Describe the setting, locations, and relevant dates, | Methods, first |
|  |  | including periods of recruitment, exposure, | to third paragraph |
|  |  | follow-up, and data collection |  |
| Participants | 6 | (*a*) Give the eligibility criteria, and the sources | Methods, first |
|  |  | and methods of selection of participants. Describe | to third paragraph |
|  |  | methods of follow-up |  |
|  |  | (*b*) For matched studies, give matching criteria | Not |
|  |  | and number of exposed and unexposed | applicable |
| Variables | 7 | Clearly define all outcomes, exposures, | Methods, third and |
|  |  | predictors, potential confounders, and effect | fourth paragraph |
|  |  | modifiers. Give diagnostic criteria, if applicable |  |
| Data sources/ | 8 | For each variable of interest, give sources of data | Results, |
| measurement |  | and details of methods of assessment | Tables 1-3 |
|  |  | (measurement). Describe comparability of |  |
|  |  | assessment methods if there is more than one |  |
|  |  | group |  |
| Bias | 9 | Describe any efforts to address potential sources | Discussion, |
|  |  | of bias | eighth paragraph |
| Study size | 10 | Explain how the study size was arrived at | Methods, first |
|  |  |  | paragraph |
| Quantitative | 11 | Explain how quantitative variables were handled | Methods, |
| variables |  | in the analyses. If applicable, describe which | Statistical analysis |
|  |  | groupings were chosen and why |  |
| Statistical methods | 12 | (*a*) Describe all statistical methods, including | Methods, |
|  |  | those used to control for confounding | Statistical analysis |

|  |  | (*b*) Describe any methods used to examine subgroups and interactions | Methods, Statistical analysis |
| --- | --- | --- | --- |
|  |  | (*c*) Explain how missing data were addressed | Not |
|  |  |  | applicable |
|  |  | (*d*) If applicable, explain how loss to follow-up | Not |
|  |  | was addressed | applicable |
|  |  | (*e* ) Describe any sensitivity analyses | Supplementary |
|  |  |  | Table S4 and S5 |
| **Results** |  |  |  |
| Participants | 13 | (a) Report numbers of individuals at each stage of | Results, |
|  |  | Study (eg, numbers potentially eligible, examined | Tables 1-3 |
|  |  | for eligibility, confirmed eligible, included in the |  |
|  |  | study, completing follow-up, and analyzed) |  |
|  |  | (b) Give reasons for non-participation at each | Not |
|  |  | stage | applicable |
|  |  | (c) Consider use of a flow diagram | Not applicable |
| Descriptive data | 14 | (a) Give characteristics of study participants (eg, | Results, |
|  |  | demographic, clinical, social) and information on | Tables 1-3 |
|  |  | exposures and potential confounders |  |
|  |  | (b) Indicate number of participants with missing | Results, |
|  |  | data for each variable of interest | Tables 1-3 |
|  |  | (c) Summarize follow-up time (eg, average and | Methods, first |
|  |  | total amount) | paragraph |
| Outcome data | 15 | Report numbers of outcome events or summary | Results, first and |
|  |  | measures over time | third paragraph |
| Main results | 16 | (*a*) Give unadjusted estimates and, if applicable, | Table S2 and S3, |
|  |  | confounder-adjusted estimates and their precision | and Figure 2 |
|  |  | (eg, 95% confidence interval). Make clear which |  |
|  |  | confounders were adjusted for and why they were |  |
|  |  | included |  |
|  |  | (*b*) Report category boundaries when continuous | Tables 1-3 |
|  |  | variables were categorized |  |
|  |  | (*c*) If relevant, consider translating estimates of | Not applicable |
|  |  | relative risk into absolute risk for a meaningful |  |
|  |  | time period |  |
| Other analyses | 17 | Report other analyses done (eg, analyses of | Table S3, S4, S5, |
|  |  | subgroups and interactions, and sensitivity | and S6 |
|  |  | analyses |  |
| **Discussion** |  |  |  |
| Key results | 18 | Summarize key results with reference to study | Discussion, first |
|  |  | objectives | paragraph |

| Limitations | 19 Discuss limitations of the study, considering sources of potential bias or imprecision. Discuss both direction and magnitude of any potential bias | Discussion,  eighth paragraph |
| --- | --- | --- |
| Interpretation | 20 Give a cautious overall interpretation of results | Discussion |
|  | considering objectives, limitations, multiplicity of |  |
|  | analyses, results from similar studies, and other |  |
|  | relevant evidence |  |
| Generalizability | 21 Discuss the generalizability (external validity) of | Discussion, last |
|  | the study results | paragraph |
| **Other information** |  |  |
| Funding | 22 Give the source of funding and the role of the | Funding |
|  | funders for the present study and, if applicable, |  |
|  | for the original study on which the present article |  |
|  | is based |  |
