# Supplementary material for: Risk factors for unfavorable outcome and impact of early post-transplant infection in solid organ recipients with COVID-19: A prospective multicenter cohort study
Source: PLoS One. 2021 Apr 29;16(4):e0250796. doi: 10.1371/journal.pone.0250796 (PMC8084252; doi:10.1371/journal.pone.0250796)
Supplement: S2 Table — (DOCX) [file pone.0250796.s004.docx]

**S2 Table. Univariable models of baseline risk factors associated with unfavorable outcome.**

|  | **Crude Odds ratio (95% CI)** | ***P*-value** |
| --- | --- | --- |
|  |  |  |
| Age ≥ 70 years | 2.88 (1.53-5.41) | .001 |
| Diabetes mellitus | 2.00 (1.08-3.69) | .03 |
| Chronic cardiopathy | 2.15 (1.13-4.11) | .02 |
| Chronic kidney disease | 1.76 (.96-3.22) | .07 |
| Dyspnea | 3.33 (1.80-6.15) | < .001 |
| Respiratory rate > 20 bpm | 4.49 (2.28-8.86) | < .001 |
| O_2_ sat < 95% | 2.01 (1.07-3.77) | .03 |
| Lymphocytes < 1 x 1000/µL | 2.04 (1.01-4.11) | .046 |
| Creatinine > 1.3 mg/dL | 2.87 (1.44-5.75) | .003 |
| Lactate dehydrogenase ≥ 300 U/L | 3.49 (1.85-6.58) | < .001 |
| C-reactive protein ≥ 100 mg/L | 2.29 (1.23-4.24) | .01 |
| D-dimer ≥ 600 ng/mL | 1.73 (.91-3.30) | .098 |
