# Supplementary material for: Risk factors for unfavorable outcome and impact of early post-transplant infection in solid organ recipients with COVID-19: A prospective multicenter cohort study
Source: PLoS One. 2021 Apr 29;16(4):e0250796. doi: 10.1371/journal.pone.0250796 (PMC8084252; doi:10.1371/journal.pone.0250796)
Supplement: S3 Table — (DOCX) [file pone.0250796.s005.docx]

**S3 Table. Univariable models of potential surrogates of immunosuppression intensity *vs.* unfavorable outcome.**

|  | **Crude Odds ratio (95% CI)** | ***P*-value** |
| --- | --- | --- |
|  |  |  |
| **Time from transplant to COVID-19 diagnosis** | | |
| Within ≤ 6 months from transplant (N = 18) | 3.28 (1.53-5.41) | **.001** |
| **Organ transplanted** | | |
| Thoracic graft (N = 48) | 1.08 (.54-2.17) | .83 |
| **Immunosuppressant dosages** | | |
| Mofetil mycophenolate ≥ 1080 mg/day (N = 35) | .65 (.28-1.51) | .32 |
| Prednisone ≥ 20 mg/day (N = 8) | 2.42 (.59-10.01) | .22 |
| **Baseline immunosuppressive regimen** | | |
| Triple therapy (N = 128) | .94 (.52-1.73) | .85 |
