# Supplementary material for: Risk factors for unfavorable outcome and impact of early post-transplant infection in solid organ recipients with COVID-19: A prospective multicenter cohort study
Source: PLoS One. 2021 Apr 29;16(4):e0250796. doi: 10.1371/journal.pone.0250796 (PMC8084252; doi:10.1371/journal.pone.0250796)
Supplement: S4 Table — (DOCX) [file pone.0250796.s006.docx]

**S4 Table. Clinical outcomes according to the type of transplant received.**

|  | **All patients 210** | **Type of transplant** | | **Statistic (df)** | **Estimated Risk (95% CI)** | ***P*-value** |
| --- | --- | --- | --- | --- | --- | --- |
|  |  | **Kidney**  **108 (51.4)** | **Others**  **102 (48.6)** |  |  |  |
| Renal complications^a^ | 83 (40.3) | 53 (49.5) | 30 (30.3) | 7.90 (1) | 2.26 (1.27-4.00) | .01 |
| Intensive care unit admission | 37 (17.6) | 21 (19.4) | 16 (15.7) | .51 (1) | 1.30 (.63-2.65) | .48 |
| Mortality | 45 (21.4) | 26 (24.1) | 19 (18.6) | .92 (1) | 1.39 (.71-2.70) | .34 |
| Graft dysfunction, at day 30 | 12 (8.3) | 11 (16.4) | 1 (1.3) | 10.72 (1) | 14.93 (1.87-119.02) | .001 |
| Graft lost, at day 30 | 5 (3.8) | 5 (7.8) | 0 (0) | 3.65 (1) | .. | .06 |
|  | | | | | | |
|  | **All patients 210** | **Type of transplant** | | **Statistic (df)** | **Estimated Risk (95% CI)** | ***P*-value** |
|  |  | **Liver**  **50 (23.8)** | **Others**  **160 (76.2)** |  |  |  |
| Intensive care unit admission | 37 (17.6) | 7 (14.0) | 30 (18.8) | .59 (1) | .71 (0.29-1.72) | .44 |
| Mortality | 45 (21.4) | 8 (16.0) | 37 (23.1) | 1.15 (1) | .63 (0.27-1.47) | .28 |
| Graft dysfunction, at day 30 | 12 (8.3) | 0 (0) | 12 (11.4) | 3.48 (1) | .. | .06 |
| Graft lost, at day 30 | 5 (3.8) | 0 (0) | 5 (5.2) | .77 (1) | .. | .38 |
|  | | | | | | |
|  | **All patients 210** | **Type of transplant** | | **Statistic (df)** | **Estimated Risk (95% CI)** | ***P*-value** |
|  |  | **Heart**  **33 (15.7)** | **Others**  **177 (84.3)** |  |  |  |
| Cardiac complications^b^ | 16 (7.6) | 3 (9.1) | 13 (7.3) | .00 (1) | 1.26 (.34-6.70) | 1.00 |
| Intensive care unit admission | 37 (17.6) | 5 (15.2) | 32 (18.1) | .16 (1) | .81 (.29-2.26) | .69 |
| Mortality | 45 (21.4) | 6 (18.2) | 39 (22.0) | .25 (1) | .79 (.30-2.04) | .62 |
| Graft dysfunction, at day 30 | 12 (8.3) | 1 (4.3) | 11 (9.1) | .12 (1) | .46 (.06-3.70) | .73 |
| Graft lost, at day 30 | 5 (3.8) | 0 (0) | 5 (4.5) | .13 (1) | .. | .72 |
|  | | | | | | |
|  | **All patients 210** | **Type of transplant** | | **Statistic (df)** | **Estimated Risk (95% CI)** | ***P*-value** |
|  |  | **Lung**  **15 (7.1)** | **Others**  **195 (92.9)** |  |  |  |
| Respiratory complications^c^ | 68 (32.4) | 5 (33.3) | 63 (32.3) | .00 (1) | 1.05 (.34-3.19) | 1.00 |
| Intensive care unit admission | 37 (17.6) | 3 (20.0) | 34 (17.4) | .00 (1) | 1.18 (.32-4.42) | 1.00 |
| Mortality | 45 (21.4) | 5 (33.3) | 40 (20.5) | .71 (1) | 1.94 (.63-5.99) | .40 |
| Graft dysfunction, at day 30 | 12 (8.3) | 0 (0) | 12 (9.2) | .46 (1) | .. | .50 |
| Graft lost, at day 30 | 5 (3.8) | 0 (0) | 5 (4.1) | .00 (1) | .. | 1.00 |
|  | | | | | | |
|  | **All patients 210** | **Type of transplant** | | **Statistic (df)** | **Estimated Risk (95% CI)** | ***P*-value** |
|  |  | **Combined**  **4 (1.9)** | **Others**  **206 (98.1)** |  |  |  |
| Intensive care unit admission | 37 (17.6) | 1 (25.0) | 36 (17.5) | .00 (1) | 1.57 (.16-15.57) | 1.00 |
| Mortality | 45 (21.4) | 0 (0) | 45 (21.8) | .19 (1) | .. | .66 |
| Graft dysfunction, at day 30 | 12 (8.3) | 0 (0) | 12 (8.4) | .00 (1) | .. | 1.00 |
| Graft lost, at day 30 | 5 (3.8) | 0 (0) | 5 (3.8) | .00 (1) | .. | 1.00 |
| ^a^New onset or exacerbation of renal insufficiency.  ^b^New onset arrhythmia, heart failure, or acute coronary event.  ^c^Need for mechanical ventilation, acute distress respiratory syndrome, empyema, or pleural effusion. | | | | | | |
