# Supplementary material for: Risk factors for unfavorable outcome and impact of early post-transplant infection in solid organ recipients with COVID-19: A prospective multicenter cohort study
Source: PLoS One. 2021 Apr 29;16(4):e0250796. doi: 10.1371/journal.pone.0250796 (PMC8084252; doi:10.1371/journal.pone.0250796)
Supplement: S5 Table — (DOCX) [file pone.0250796.s007.docx]

**S5 Table. Description of patients suffering from graft dysfunction at day 30 (n=12).**

| **Identification, demographics, type of transplant (dd.mm.yy), and presence of pneumonia** | **Previous rejection**  **history** | **Hospital admission** | **Intensive care unit admission** | **Graft loss** | **Death** | **Specific treatment for COVID-19** | **Baseline regimen** | **Handle of immunosuppression** |
| --- | --- | --- | --- | --- | --- | --- | --- | --- |
| **1.** 59-year-old male, kidney transplant (01.01.94), pneumonic | No | **Yes** | No | **Yes** | No | LPV/r + HCQ + TCZ + IFN + MPS | Pred + Tac + MMF | End of Tac and MMF. Reduction of Pred |
| **2.** 79-year-old male, kidney transplant (24.02.08), pneumonic | No | **Yes** | No | No | No | HCQ + AZM | Pred + Tac + SRL/EVR | End of Tac and SRL/EVR. Pred kept. |
| **3.** 57-year-old male, kidney transplant (13.06.10), pneumonic | No | **Yes** | **Yes** | **Yes** | **Yes** | LPV/r + HCQ + AZM | Pred + Tac + SRL/EVR | End of Tac and SRL/EVR. Pred kept. |
| **4.** 38-year-old male, **heart** transplant (09.07.11), pneumonic | **Yes, chronic** | **Yes** | No | No | No | HCQ | Pred + Tac + MMF | End of MMF. Tac and Pred kept. |
| **5.** 48-year-old male, kidney transplant (30.07.11), pneumonic | No | **Yes** | No | No | No | HCQ + AZM | Pred + Tac + SRL/EVR | End of Tac and SRL/EVR. Pred kept. |
| **6.** 73-year-old male, kidney transplant (12.03.14), pneumonic | No | **Yes** | **Yes** | **Yes** | **Yes** | LPV/r + HCQ + TCZ | Pred + Tac + MMF | End of Tac and MMF. Pred kept. |
| **7.** 56-year-old male, kidney transplant (16.09.14), pneumonic | No | **Yes** | **Yes** | No | **Yes** | LPV/r + HCQ + TCZ | Pred + Tac + SRL/EVR | End of Tac and SRL/EVR. Pred kept. |
| **8.** 50-year-old male, kidney transplant (08.05.16), pneumonic | No | **Yes** | No | No | No | HCQ + TCZ + AZM | Pred + Tac + SRL/EVR | End of Tac and SRL/EVR. Pred kept. |
| **9.** 45-year-old female, kidney transplant (20.12.16), pneumonic | No | **Yes** | No | **Yes** | No | LPV/r + HCQ | Pred + Tac + MMF | End of Tac and MMF. Pred kept. |
| **10.** 39-year-old male, kidney transplant (24.03.17), pneumonic | No | **Yes** | No | No | No | HCQ | Pred + Tac + SRL/EVR | End of Tac and SRL/EVR. Pred kept. |
| **11.** 61-year-old female, kidney transplant (18.02.20), **non-pneumonic** | **Yes, acute** | **Yes** | No | **Yes** | No | HCQ | Pred + Tac + MMF | **No changes** |
| **12.** 40-year-old female, kidney transplant (08.03.20), pneumonic | No | **Yes** | No | No | No | **None** | Pred + Tac + MMF | End of MMF. Tac and Pred kept. |
| Abbreviations: LPV/r, lopinavir/ritonavir; HCQ, hydroxychloroquine; TCZ, tocilizumab; IFN, interferon; MPS, methylprednisolone; AZM, azithromycin; Pred, prednisone; Tac, tacrolimus; MMF, mofetil mycophenolate; SRL/EVR, sirolimus/everolimus. | | | | | | | | |
