# Supplementary material for: Risk factors for unfavorable outcome and impact of early post-transplant infection in solid organ recipients with COVID-19: A prospective multicenter cohort study
Source: PLoS One. 2021 Apr 29;16(4):e0250796. doi: 10.1371/journal.pone.0250796 (PMC8084252; doi:10.1371/journal.pone.0250796)
Supplement: S6 Table — (DOCX) [file pone.0250796.s008.docx]

**S6 Table. Description of patients with COVID-19 acquisition during the first month posttransplant (n=6).**

| **Identification, demographics, type of transplant (dd.mm.yy), and presence of pneumonia** | **Previous rejection**  **history** | **Hospital admission** | **Intensive care unit admission** | **Graft dysfunction at day 30** | **Death** | **Specific treatment for COVID-19** | **Baseline regimen** | **Handle of immunosuppression** |
| --- | --- | --- | --- | --- | --- | --- | --- | --- |
| **1.** 40-year-old female, kidney transplant (08.03.20), pneumonic | No | **Yes** | No | **Yes (but no graft loss)** | No | **None** | Pred + Tac + MMF | End of MMF. Tac and Pred kept. |
| **2.** 61-year-old female, kidney transplant (18.02.20), **non-pneumonic** | **Yes, acute** | **Yes** | No | **Yes (with graft loss)** | No | HCQ | Pred + Tac + MMF | **No changes** |
| **3.** 70-year-old male, kidney transplant (20.03.20), **non-pneumonic** | No | **Yes** | No | No | No | HCQ | Pred + Tac | Reduction of Tac. Pred kept. |
| **4.** 43-year-old male, **heart** transplant (02.03.20), pneumonic | No | **Yes** | **Yes** | No | No | LPV/r + HCQ | Pred + Tac + MMF | **No changes** |
| **5.** 59-year-old female, **first week** post **heart** transplant (30.03.20), pneumonic | No | **Yes** | **Yes** | No | **Yes** | LPV/r + HCQ + TCZ | Pred + Tac + MMF | End of Tac and MMF. Pred kept. |
| **6.** 72-year-old female, **first week** post kidney transplant (27.03.20), pneumonic | No | **Yes** | **Yes** | No | **Yes** | HCQ | Pred + MMF | Reduction of MMF. Pred kept. |
| Abbreviations: LPV/r, lopinavir/ritonavir; HCQ, hydroxychloroquine; TCZ, tocilizumab; IFN, interferon; MPS, methylprednisolone; AZM, azithromycin; Pred, prednisone; Tac, tacrolimus; MMF, mofetil mycophenolate; SRL/EVR, sirolimus/everolimus. | | | | | | | | |
