# Supplementary material for: Risk factors for unfavorable outcome and impact of early post-transplant infection in solid organ recipients with COVID-19: A prospective multicenter cohort study
Source: PLoS One. 2021 Apr 29;16(4):e0250796. doi: 10.1371/journal.pone.0250796 (PMC8084252; doi:10.1371/journal.pone.0250796)
Supplement: Attachment — Submitted filename: Response to Reviewers_10-3-21.docx [file pone.0250796.s010.docx]

**Reviewer # 1:**

The manuscript is well written. I have one question: in the analysis, there were 10 patients that were discharged and readmitted during the study period. For those 10 patients, how many days between their discharge and readmission? How many of them yield unfavorable outcomes? Will the results change significantly if including their 2nd hospital admission episode in the logistic regression? Because the p-value for lactate dehydrogenase >= 300 is just slightly smaller than 0.05 and its 95% CI is also very close to 1, it is possible that a slight change in the data would change the conclusion. The same for Lymphocytes < 1, p-value close to 0.05 and 95% CI close to 1.

*Thanks to the Reviewer by his positive appreciation of the manuscript. Please see below the answers to the two questions.*

*Regarding the question on the 10 patients readmitted during the study period: i) the median of days from discharges to readmissions was 7 days (range 1 to 19); therefore, we consider the readmission as part of the same episode of SARS-CoV-2 infection; ii) only one patient needed intensive care after readmission, but he also required it during his first admission; and the 10 patients were discharged alive after the readmissions; iii) in summary, there was no change in the number of unfavorable outcome (a composite of ICU admission and/or death) in the cohort of the 210 solid organ transplantation recipients.*

Another question is: some variables were “dichotomized based on normal ranges” in the analysis. I would like to know if these thresholds were determined by authors or based on some well-established or well-accepted criteria.

*To examine factors associated with unfavorable clinical outcome, we dichotomized quantitative variables based on normal ranges, and after addressing their effect as continuous variables. The cut-offs used to dichotomize the variables were chosen based both in the normal values and in the cut-offs associated with unfavorable outcome when we analyzed the general population (Salto-Alejandre S et al. J Infect 2021 Feb;82(2):e11-e15. doi: 10.1016/j.jinf.2020.09.023. Epub 2020 Sep 25).*

*Following the Reviewer question, to clarify it we have changed the sentence of the previous manuscript as follows: “… quantitative variables were dichotomized based on normal ranges* *and in the cut-offs associated with unfavorable outcome in the general population [28], after addressing their effect as continuous.” (lines 155-157 of the revised manuscript). We have added this new reference [28] in the manuscript.*

**Reviewer # 2**

This is an interesting paper. The study is well designed and with proper statistical analysis of the data.  However, the manuscript is poorly written and needs extensive editing to correct for typos and grammatical errors. I have few minor comments.

*Thanks to the Reviewer by his positive appreciation of the design and the data analysis.*

Line 159: Replace confusion by confounding

*We have replaced confusion by confounding (line 159 of the revised manuscript), as requested by the Reviewer.*

Line 311. Clarify hypothetically

*Besides the main objective of the study, we wanted to supply descriptive data on patients with possible poorer outcome, because of graft dysfunction (12 patients) or developing COVID-19 in the period of maximal immunosuppressive therapy as it is the first month after receiving the transplant (6 patients). In this regard “hypothetically” was used as synonymous of “possible”. To clarify it, we have substituted the sentence “Two subgroups of the study population were considered, hypothetically, of higher risk” for “Two subgroups of the study population were considered of possible higher risk” (line 309 of the revised manuscript).*

Line 327: Spell out the “abovementioned unfavorable outcomes”

*Following the Reviewer request we have changed it to “ICU admission and/or death” (lines 318-319 of the revised manuscript).*

Line 329: What is severity risk factor?

*Severity was used as synonymous of unfavorable outcome. To clarify it, we have changed this sentence as follows: “… as a risk factor for unfavorable outcome.” (lines 319-320 of the revised manuscript).*

Line 349:   Replace “cardiopathy” with “cardiomyopathy”

*Following this request, we have replaced “cardiopathy” with “cardiomyopathy” (line 340 of the revised manuscript).*

Line 351: Add “and” after “included, “

*As requested, we have added “and” (line 342 of the revised manuscript).*

Line 377: Replace “proved” with “showed”.

*As requested, we have replaced “proved” with “showed” (line 368 of the revised manuscript).*

Line 385: Replace “accurate” with “strong”.

*As requested, we have replaced “accurate” with “strong” (line 376 of the revised manuscript).*
